# Assessing age-related gray matter decline with voxel-based morphometry depends significantly on segmentation and normalization procedures

**DOI:** 10.3389/fnagi.2014.00124

**Published:** 2014-06-23

**Authors:** Dorothée V. Callaert, Annemie Ribbens, Frederik Maes, Stephan P. Swinnen, Nicole Wenderoth

**Affiliations:** ^1^Movement Control and Neuroplasticity Research Group, Department of KinesiologyKU Leuven, Belgium; ^2^CNRS, INCIA, UMR 5287, University of BordeauxTalence, France; ^3^Department of Electrical Engineering – ESAT – PSI & iMinds - Future Health DepartmentKU Leuven, Belgium; ^4^Neural Control of Movement Laboratory, Health Sciences and TechnologyETH Zurich, Switzerland

**Keywords:** ageing, gray matter, atrophy, brain mapping, volumetry, MRI

## Abstract

Healthy ageing coincides with a progressive decline of brain gray matter (GM) ultimately affecting the entire brain. For a long time, manual delineation-based volumetry within predefined regions of interest (ROI) has been the gold standard for assessing such degeneration. Voxel-Based Morphometry (VBM) offers an automated alternative approach that, however, relies critically on the segmentation and spatial normalization of a large collection of images from different subjects. This can be achieved via different algorithms, with SPM5/SPM8, DARTEL of SPM8 and FSL tools (FAST, FNIRT) being three of the most frequently used. We complemented these voxel based measurements with a ROI based approach, whereby the ROIs are defined by transforms of an atlas (containing different tissue probability maps as well as predefined anatomic labels) to the individual subject images in order to obtain volumetric information at the level of the whole brain or within separate ROIs. Comparing GM decline between 21 young subjects (mean age 23) and 18 elderly (mean age 66) revealed that volumetric measurements differed significantly between methods. The unified segmentation/normalization of SPM5/SPM8 revealed the largest age-related differences and DARTEL the smallest, with FSL being more similar to the DARTEL approach. Method specific differences were substantial after segmentation and most pronounced for the cortical structures in close vicinity to major sulci and fissures. Our findings suggest that algorithms that provide only limited degrees of freedom for local deformations (such as the unified segmentation and normalization of SPM5/SPM8) tend to overestimate between-group differences in VBM results when compared to methods providing more flexible warping. This difference seems to be most pronounced if the anatomy of one of the groups deviates from custom templates, a finding that is of particular importance when results are compared across studies using different VBM methods.

## Introduction

The human brain undergoes continuous structural changes due to development and aging. Post mortem studies have shown that even healthy aging is accompanied by notable cortical atrophy and loss of brain weight from the sixth life decade onwards (Skullerud, 1985). This brain tissue loss is further accelerated by most neurodegenerative or neuropsychiatric disorders such that magnetic resonance imaging (MRI) in combination with morphometry offers an interesting diagnostic tool for differentiating between healthy and pathological brain changes (for an overview, see Raz and Rodrigue, 2006; Mueller et al., 2012a,b). One common approach is to evaluate the volume of gray matter (GM), white matter (WM) and cerebrospinal fluid (CSF) within structures of interest determined through manual delineation. Even though this approach has long been considered the “gold standard” it is very labor-intensive and requires high anatomical expertise such that investigations were frequently restricted to a limited set of predefined anatomical regions of interest (ROI). Alternatively, semi-automated procedures were incorporated for detecting region boundaries and reducing observer-dependence (Mu et al., 1999; Jernigan et al., 2001; Tisserand et al., 2002; Raz et al., 2003; Allen et al., 2005a; Walhovd et al., 2005, 2010; Greenberg et al., 2008; Hasan and Pedraza, 2009). During the last decade, volumetry of predefined brain structures has been complemented by whole-brain, voxel-based methods like voxel-based morphometry (VBM; Good et al., 2001; Ashburner and Friston, 2000, 2001; Tisserand et al., 2004; Gonoi et al., 2010), or estimating voxel-based cortical thickness (Hutton et al., 2008). Particularly VBM has been frequently used for investigating GM decline in the context of neurodegenerative pathologies (e.g., Good et al., 2002; Testa et al., 2004; Chetelat et al., 2005; Giuliani et al., 2005; Douaud et al., 2006; Keller and Roberts, 2008; Davies et al., 2009; Ibarretxe-Bilbao et al., 2009; Ferreira et al., 2011) and healthy ageing (Good et al., 2001; Van Laere and Dierckx, 2001; Resnick et al., 2003; Tisserand et al., 2004; Grieve et al., 2005; Jernigan and Gamst, 2005; Lemaıtre et al., 2005; Lehmbeck et al., 2006; Fjell et al., 2009; Kalpouzos et al., 2009; Kennedy et al., 2009; Ziegler et al., 2012). In a nutshell, VBM assumes that each voxel of a T1-weighted high resolution MR image contains a mixture of GM, WM, and CSF. The image is segmented to yield the content or probability of each tissue class at the voxel level. After transforming each individual brain image into the same stereotactic space (thus correcting for global differences in position, size, and shape across individuals) these voxel-based tissue probabilities can be statistically compared between different populations to determine local alterations of brain structure.

VBM has been frequently applied to show that healthy aging is accompanied by GM tissue loss. Even though changes are distributed across the brain there is particular vulnerability with respect to the frontal lobes (Good et al., 2001; Van Laere and Dierckx, 2001; Tisserand et al., 2004; Grieve et al., 2005; Lehmbeck et al., 2006; Kennedy et al., 2009; Kalpouzos et al., 2009). This general finding was further confirmed by manual delineation and semi-automated ROI-based studies (Jernigan et al., 2001; Raz et al., 2004; Allen et al., 2005a; Walhovd et al., 2005, 2010). However, it is worth noting that when VBM and manual delineations were applied to the same data set, results were only partly congruent. Investigating (pre)frontal subregions, Tisserand et al. (2002) consistently identified pronounced age-related GM reduction in fronto-lateral areas with both VBM and manual volumetry. Method-dependent differences, however, were revealed for orbital portions: whereas these exhibited age-effects when using manual delineation, the voxel-based approach identified only cingulate subregions. Allen et al. (2005b) assessed VBM against manual volumetry for insular cortex and detected a different rate of age-induced degeneration: while VBM indicated the pace of insular decline to exceed that of frontal cortex, the manual delineation results instead attributed a slower pattern of decline. In a seminal study, Kennedy et al. (2009) compared VBM to manual tracings of predefined ROIs in a large sample of 200 subjects. They demonstrated that VBM might reveal reliable information when aggregated within meaningful anatomical regions. However, when interpreted at the voxel level, VBM tended to overestimate age related GM differences, particularly for voxels bordering WM or CSF.

One important factor that may contribute to inconsistencies across studies is that VBM appears to be very sensitive to small methodological variations. Thus, even minor differences in spatial transformations or smoothing procedures might yield widely divergent results as has been demonstrated for longitudinal VBM studies in GM changes (Thomas et al., 2009; Thomas and Baker, 2013) and the analysis of WM structures (Jones et al., 2007). Here we revisit this issue for the comparison of brain structure and, particularly, GM volume between different age groups. The motivation for our study is that image analysis methods are constantly modified and improved, while it is rarely the case that published results are reanalyzed using the new algorithms. VBM relies critically on the segmentation and normalization of the structural images and different algorithms have been implemented to solve these problems. Two of the most frequently used software packages are SPM (Wellcome Trust Centre for Neuroimaging, London, UK) and FSL (Smith et al., 2004; Douaud et al., 2006), and both offer VBM data processing pipelines. Within SPM two versions are available: one uses the unified segmentation and normalization routines as implemented in standard SPM5/SPM8. This method determines GM, WM, and CSF tissue classes in an iterative procedure that warps the T1- weighted images of each individual subject to the ICBM152 space (i.e., MNI space) and segments them using SPM5/SPM8 standard prior tissue probability maps (Ashburner and Friston, 2005). One disadvantage of the SPM5/SPM8 procedure is that local, non-linear transformations are rather limited because warping is achieved through a restricted set of spatial basis functions (Ashburner and Friston, 2004) that are regularized by minimizing the bending energy of the deformation fields. In the widely used SPM8 release a new segmentation procedure has been implemented which determines additional tissue classes to reduce misclassification and uses prior tissue probability maps based on a large sample with a broad age range. Moreover, an alternative normalization procedure, known as DARTEL, uses a diffeomorphic algorithm to warp individual subject images into a common space that is defined in an iterative procedure to determine one common template optimized for the population under study (Ashburner, 2007; Ashburner and Friston, 2009). DARTEL is believed to allow more local, non-linear deformations than the standard SPM5/SPM8 normalization and to reveal better results in populations with deviant anatomy. FSL uses a segmentation procedure independent of prior information to determine the different tissue classes. Instead it applies a hidden Markov random field model together with an associated Expectation-Maximization algorithm to associate each intensity value from the anatomical image with a specific mixture of GM, WM and CSF probabilities (Zhang et al., 2001). Normalization is achieved by combining affine with nonlinear registration (Andersson et al., 2007a,b). The use of a b-spline representation of the registration warp field (Rueckert et al., 1999) allows application of highly local deformations in order to match individual anatomy to a common template.

Here we address the question of how far methodological differences regarding the segmentation (i.e., partitioning anatomical images in GM, WM, and CSF probability maps) and normalization of MRI images (i.e., transforming the individual anatomical images to a template) influence VBM results. We use the aging brain as a model for a significant reduction in GM volume that has been consistently demonstrated by a large number of previous studies using various methods. Moreover, we compared two groups (young vs. elderly subjects) at the extreme spectrum of the age range to compare how the different methods influence the effect size of age-related structural changes.

In addition to those software packages freely available, we complemented the VBM results with an automated ROI approach using an independent algorithm to determine volumetric measurements within predefined anatomical structures (D'Agostino et al., 2003, 2006a,b; Stiers et al., 2005; Machilsen et al., 2007).

## Materials and methods

### Participants

Thirty-nine subjects, 21 young (23 ± 1.7 years; range 20–27; 8 male/13 female) and 18 elderly (66.2 ± 3.4 years; range 62–73; 9 male/9 female), participated in the study. All subjects were strongly right-handed, as determined by the Edinburgh handedness questionnaire (Oldfield, 1971) and none of the participants reported a history of neurological disease, sensorimotor dysfunction or used psycho- or vasoactive medication. General functions were assessed using the Mini Mental State examination and all scores ranged within normal limits (score ≥26). Participants were informed about the experimental procedures and provided written informed consent. The informed consent and the study design were approved by the local Ethics Committee of Biomedical Research at KU Leuven, in accordance with the ethical standards laid down in the 1964 Declaration of Helsinki.

### MRI protocol

Whole-brain T1-weighted images were collected on a 3-T Intera MRI scanner (Philips, Best, Netherlands), using a six element SENSE head coil (MRI Devices Corp., Waukesha, WI). Each volume consisted of 182 slices, with echo time (TE) 4.6 ms, repetition time (TR) 9.68/9.58 ms, inversion time (TI) 1100 ms, field of view (FOV) 250 mm, matrix 256 × 256, in plane resolution of 0.98 × 0.98 mm^2^, slice thickness 1.2 mm, and SENSE factor 2. None of the participants exhibited abnormalities in brain structure.

### Image processing

We analyzed the data with four different approaches: (1) the unified segmentation/normalization algorithm “SPM8 segment” as implemented in SPM5/SPM8 (subsequently called SPM VBM), (2) DARTEL using the “SPM8 new segment” and normalization procedure implemented in SPM8 (subsequently called DARTEL VBM), (3) tools of the FSL package (subsequently called FSL VBM), and (4) a procedure combining Intensity-Based Segmentation and atlas-to-image Non-Rigid Registration (IBSNRR) with volumetry for predefined ROIs. For each method, we followed the procedures advised by the software developers to generate GM, WM, and CSF segments in individual subject space. For the first three methods we also determined a set of warping parameters defining the transformation from individual subject space to MNI ICBM152 template space.

### SPM VBM

**Figure 1:**
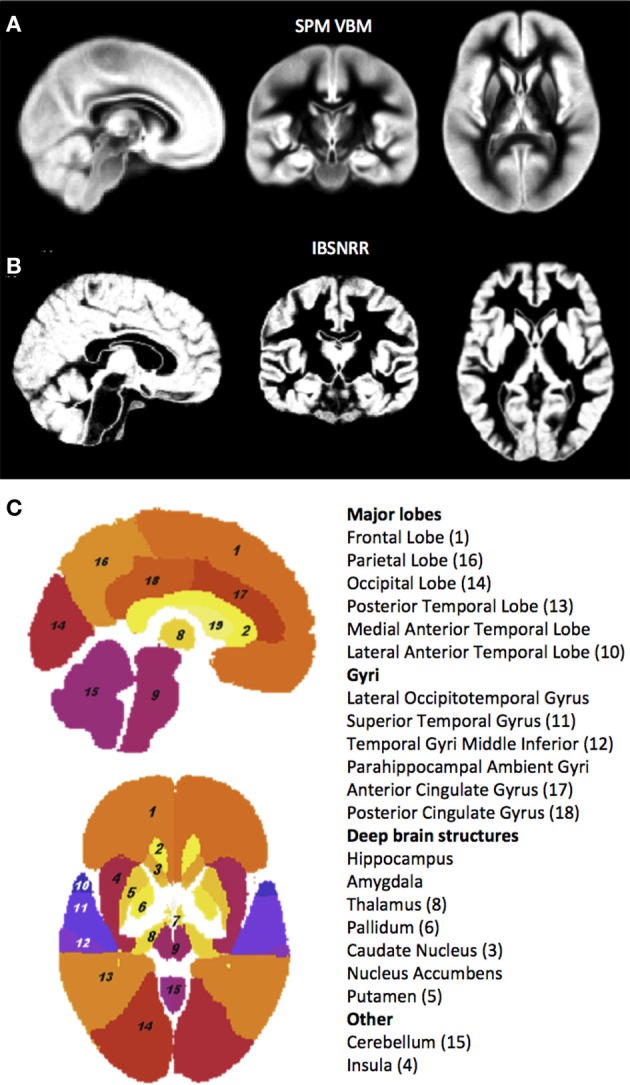
**GM probability maps used by the unified segmentation and normalization procedure of SPM5/SPM8 (A) and the IBSNRR approach (B)**. Note that the IBSNRR atlas provides much more anatomical detail than the GM probability map in **(A)**. Labels of the IBSNRR atlas are shown in **(C)**. Anatomical structures are numbered in accordance to the label names (numbers are provided in brackets).

All SPM analyses were performed with SPM8 (v4667, Wellcome Trust Centre for Neuroimaging, London, UK) run in Matlab Version 7.6.0.324 (R2008a). Segmentation of the images was performed by the unified segmentation routine “SPM8 segment” as implemented in SPM5/SPM8 (Ashburner and Friston, 2005). Briefly, this procedure segments the T1-weighted image in GM, WM, and CSF tissue classes, accomplished by iterative registration to ICBM152 space (i.e., MNI template space) and segmentation using SPM5/SPM8 standard prior tissue probability maps. It is worth noting, however, that the standard SPM5/SPM8 probability maps were derived based on a sample of young adults only and, thus, might introduce a bias when examining age effects (Figure 1A). For each participant, the segmentation procedure yields GM, WM, and CSF probability maps in individual subject space as well as deformation fields describing the transformation of the tissue priors to the native subject images. The GM probability maps were warped to standard ICBM152 space by applying the inverse of the deformation fields determined during segmentation.

### DARTEL VBM

Segmentation was performed using the unified segmentation (Ashburner and Friston, 2005) as implemented in the “New segment” function of SPM8. This segmentation determines more tissue classes than standard SPM5/SPM8 including GM, WM, CSF, soft tissue, skull, and out-of-brain regions of the image. Prior probability maps were derived from a large sample of healthy adults across the lifespan (Good et al., 2001) that are available in ICBM152 space. First, a customized template was created based on the GM segmentations using a diffeomorphic method known as DARTEL (Ashburner, 2007; Ashburner and Friston, 2009). DARTEL uses an iterative process to progressively refine warping parameters describing the transformation from individual subject space to a common space. During this procedure flow fields were calculated and subsequently applied to the native GM image of each subject. Next, the affine transformation from the customized template space to ICBM152 space was calculated and applied to the individual GM segmentations.

### FSL VBM

Using the FSL 4.1.5 tools (Smith et al., 2004), all individual T1 weighted images were brain-extracted using BET (Smith, 2002) and visually inspected. Then tissue-type segmentation was carried out using FAST v4.1 (Zhang et al., 2001). Importantly, FAST does not use prior information to determine the different tissue classes but applies a hidden Markov random field model together with an associated Expectation-Maximization algorithm to associate each intensity value from the anatomical image with a specific mixture of GM, WM, and CSF probabilities. Then a study specific template was created. Therefore, the resulting GM probability images were normalized to ICBM152 standard space using the standard FSL template and the affine registration tool FLIRT (Jenkinson and Smith, 2001; Jenkinson et al., 2002), followed by nonlinear registration executed by FNIRT (Andersson et al., 2007a,b), which uses a b-spline representation of the registration warp field (Rueckert et al., 1999). The resulting images were flipped and averaged to create a symmetric, study-specific template. In a second iteration, the native GM probability maps were then non-linearly re-registered to the customized template.

### IBSNRR: intensity-based segmentation and non-rigid registration

The general idea of IBSNRR is that tissue class priors of anatomical ROIs are predefined by a statistical brain atlas that gets transformed to individual subject space. As a result the shape of each structural ROI is adjusted to individual anatomy and GM, WM, CSF and non-brain tissue probability maps are obtained within predefined structures in native subject space (Stiers et al., 2005; Machilsen et al., 2007). Subsequently, tissue volumes are quantified within each ROI. As such, this method implements automated ROI volumetry rather than a VBM approach. IBSNRR uses tissue class priors for segmentation derived from a statistical brain atlas constructed from 20 manually labeled brain images of 10 males and 10 females (median age 31 years) acquired at the Imperial College, Hammersmith Hospital (London), at an average voxel size of 0.937 mm^3^ (Hammers et al., 2002, 2007; Wang et al., 2005). This atlas consists of a T1-weighted image, with corresponding probability maps for global tissue classes of GM, WM, and CSF (Figure 1B) and for 49 predefined anatomical structures (Figure 1C). Initial affine atlas-to-image registration was performed based upon the atlas mean gray-scale image and the T1-weighted subject images, using mutual information (Maes et al., 1997). Next, segmentation occurred within a unified framework that alternates between an intensity-based segmentation step using a Gaussian mixture model (analogous to Van Leemput et al., 1999) and a non-rigid registration step that matches the tissue class maps of the atlas to the segmented subject images using a viscous-fluid model (D'Agostino et al., 2003, 2006a,b). This algorithm yields atlas-to-image deformation fields allowing for local non-linear transformations that outperform direct atlas-to-image registrations. By applying these deformation fields to the tissue probability maps of the atlas anatomical structures, a segmentation solution was obtained. Segmentation results were visually inspected by two independent observers and no major misclassifications were detected.

### Data analysis and statistics

#### Whole brain volumes

All four methods yielded GM, WM, and whole brain CSF probability maps in native space. From these images, total GM, WM, and CSF volumes were determined by thresholding each tissue probability map at *p* ≥ 0.2, multiplying the tissue probability with the volume of the voxel and integrating these values across the brain. Subsequently, these volumes were subjected to an analysis of variance for repeated measurements (repeated measures ANOVA) with the between factor Age (young, old) and Meth (SPM, DARTEL, FSL, IBSNRR). One important confound for between-group analyses of brain volume is the average head size of the participants. For example, if our elderly subject sample had larger brains on average, it would not be surprising if they also had more GM volume, since brain size and GM volume are expected to be proportionally related. To prevent this potential bias we estimated the intracranial volume (ICV) for each participant. We aimed to have an ICV estimate independent of the 4 evaluated segmentation methods and therefore used the dilated brain mask of the ICBM152 template, available from the FSL software package. This mask was transformed to native subject space based on the inverted, non-linear warping parameters derived from the FSL normalization procedure and visually inspected (see Supplementary Table 1 for additional justification and analyses motivating this specific approach). Subsequently, ICVwas estimated by integrating the volume across all non-zero voxels of the mask and ICV was used when whole brain GM, WM, and CSF volumes were statistically compared either by adding it as a covariate of no interest, or by proportionally scaling GM, WM, and CSF volumes to the ICV volume [i.e., GM (%) = GM (l)/ICV (l) ^*^100] prior to the statistical comparisons.

Next we asked whether the distribution of GM, WM, and CSF probabilities would differ between methods. Distributions were determined for each method, age group and tissue class by counting voxels with *p* ≥ 0.05 and *p* ≤ 1 in bins of 0.05, and expressing them as a percentage of ICV. Repeated measures ANOVAs with the factor Bin (0.05–0.1, …., 0.95–1) and Meth (SPM, DARTEL, FSL, IBSNRR) were calculated separately for each tissue class and Tukey *post-hoc* tests were used to test for method specific differences within each bin. We then quantified age specific differences in the GM probability distribution. Therefore, the mean volume of the young subjects was calculated for each bin and the GM volume of each elderly participant was expressed as a fraction of this value. These values were subjected to a repeated measures ANOVA with the within factors Bin and Meth.

For all analyses alpha was set to 0.05 and Tukey *post-hoc* tests were applied when appropriate. All statistical analyses above were performed with Statistica 10 (StatSoft Inc., Tulsa USA).

#### GM volumetry: interactions between age and segmentation method

GM segmentations derived by each of the four methods were thresholded at *p* ≥ 0.2, a minimum probability value often recommended for SPM VBM analyses. We wanted to quantify whether the segmentation method affects age specific GM differences either with respect to the localization or the size of the effect. Two analyses were carried out to answer this question. First, the thresholded GM probability maps derived by each of the four methods were normalized to ICBM152 space by applying *the same* non-rigid transformation determined by SPM8 to all images of one subject. Normalized images were resampled to 2 × 2 × 2 mm voxels using a nearest neighbor interpolation method and no modulation for resizing voxels was applied. Next the normalized GM probability maps were smoothed with a 10 mm FWHM Gausian kernel and subjected to a repeated measures ANOVA in SPM with the between factor Age (young, old) and the within factor Meth (SPM, DARTEL, FSL, ISBNRR). Finally we calculated all Age × Meth interaction effects and the resulting statistical parametric maps (Friston et al., 1994) were thresholded at *p*_uncor_ < 0.001 (i.e., uncorrected for multiple comparisons) and at *p*_FWE_ < 0.05 (i.e., after applying family wise error correction). This analysis revealed where in the brain age-related GM differences depend significantly on the segmentation method. Note however, that normalization into the common ICBM 152 space might have been suboptimal for comparing young vs. elderly subjects.

Therefore, we performed a second complementary analysis determining mean GM probability within 21 bilateral ROIs derived by the IBSNRR method (for a list of these areas see Supplementary Table 1). More specifically, GM probability values were multiplied with the voxel volume and these values were integrated across all voxels within a given ROI and with *p* > 0.2 and subsequently divided by ICV. This procedure was performed for each individual and each method. Next, for each ROI and each method, data were *z*-transformed (i.e., the mean across all subjects was subtracted and this value was divided by the standard deviation). This allows the identification of the effect size of age specific differences while removing variability introduced by the segmentation method *per se*. For each ROI and each segmentation method, *z*-transformed GM volume of the young and the elderly were subjected to independent *t*-tests and a Bonferroni correction was applied to the resulting *p*-values. Additionally, Cohen's d effect sizes were calculated.

#### GM VBM: the influence of segmentation and normalization methods on age specific GM differences

Finally, GM probability maps derived from SPM5/SPM8, DARTEL, and FSL segmentation were thresholded at *p* > 0.2 and transformed to ICBM152 space by applying the warpings as obtained with SPM5/SPM8, DARTEL and FSL normalization, respectively (resulting in nine normalized GM data sets in total). Additionally, a modulation step was included to keep the total GM amount constant despite local expansion or contraction introduced by image normalization. For each of the 3 segmentation × 3 normalization data sets, a separate second level model was specified in SPM, calculating independent *t*-tests between young and elderly subjects while GM data were proportionally scaled for ICV. The contrast young > elderly was calculated and thresholded at *p*_uncor_ < 0.001 as well as *P*_FWE_ < 0.05. These results were qualitatively compared to describe differences introduced by the various normalization procedures.

## Results

### GM, WM, and CSF: cross-method comparison of detecting ageing effects on overall volumes

GM, WM, CSF, and ICV are shown separately for the young and the elderly group in Table 1. For all tissue classes, there was a significant Age × Meth interaction irrespective of whether ICV was used as a covariate (upper part of the Table 1) or as proportional scaling factor (lower part of Table 1). More specifically, GM volumes were generally smaller in the elderly than in the young subjects. However, the extent of this age specific effect differed across methods. Testing the Age × Meth interaction pairwise between methods (and applying a Bonferroni correction to the *p*-values) revealed that SPM segmentation resulted in significantly larger age related differences in GM volume than all other methods (*p* < 0.005). By contrast, DARTEL segmentation resulted in significantly smaller age effects than all other methods (*P* < 0.005). These results were driven by the fact that SPM yielded exceptionally small GM volumes for the old group.

**Table 1:** **Whole brain volumes (Vol) estimated based on tissue segmentations derived from SPM, DARTEL, FSL, or ISBNRR**.

	**SPM**		**DARTEL**		**FSL**		**IBSNRR**		**ANOVA**	
	**Vol (ml)**		**Vol (ml)**		**Vol (ml)**		**Vol (ml)**		**Group × Meth**	
	**Young**	**Elderly**	**Young**	**Elderly**	**Young**	**Elderly**	**Young**	**Elderly**	***F*_(3, 108)_**	***p***
GM	741 ± 78.1	625 ± 53.4	688 ± 57.0	653 ± 59.9	627 ± 58.7	563 ± 41.3	758 ± 78.3	682 ± 61.8	31.75	<0.0001
WM	450 ± 39.5	462 ± 65.3	489 ± 42.8	489 ± 57.7	522 ± 47.6	518 ± 63.0	415 ± 38.3	410 ± 85.6	3.69	<0.05
CSF	372 ± 77.0	479 ± 78.0	255 ± 18.1	253 ± 24.2	281 ± 30.5	315 ± 33.3	181 ± 17.5	215 ± 27.2	10.92	<0.001
	**%ICV**		**%ICV**		**%ICV**		**%ICV**		**Group × Meth**	
	**Young**	**Elderly**	**Young**	**Elderly**	**Young**	**Elderly**	**Young**	**Elderly**	***F*_(3, 111)_**	***p***
GM	43 ± 2.4	36 ± 1.7	40 ± 0.9	38 ± 1.0	36 ± 1.4	33 ± 1.4	44 ± 1.4	40 ± 1.0	32.21	<0.0001
WM	26 ± 0.7	27 ± 1.8	28 ± 0.7	29 ± 1.1	31 ± 1.1	30 ± 1.4	24 ± 0.9	25 ± 1.9	3.6	<0.05
CSF	22 ± 4.6	28 ± 3.6	13 ± 0.7	14 ± 0.6	16 ± 1.2	18 ± 1.2	10 ± 0.7	13 ± 1.1	10.6	<0.001

Volumes are expressed in ml (upper part) or as a percentage of the ICV (lower part). Statistical results are reported in the rightmost columns. Post-hoc analyses comparing the Group × Method interaction pairwise between methods are reported in the text.

WM volumes were estimated much more similarly across methods. Small, but significant differences, were revealed because SPM segmentation indicated a larger increase in WM volume for the elderly as compared to the young than in the other methods. Accordingly, the age effect was significantly larger for SPM than for DARTEL or FSL segmentations (*p* < 0.05).

CSF volumes were larger in the elderly than in the young group, however this age effect was much more pronounced when estimated with SPM than with all other methods (*p* < 0.0003) because CSF estimates yielded by SPM were exceptionally large in the elderly.

In summary, we found large differences across segmentation methods when quantifying the effect of ageing on changes in overall tissue class volumes. With respect to GM, SPM tended to over-estimate age specific differences, while DARTEL tended to under-estimate these structural changes when compared to the other methods.

**Figure 2:**
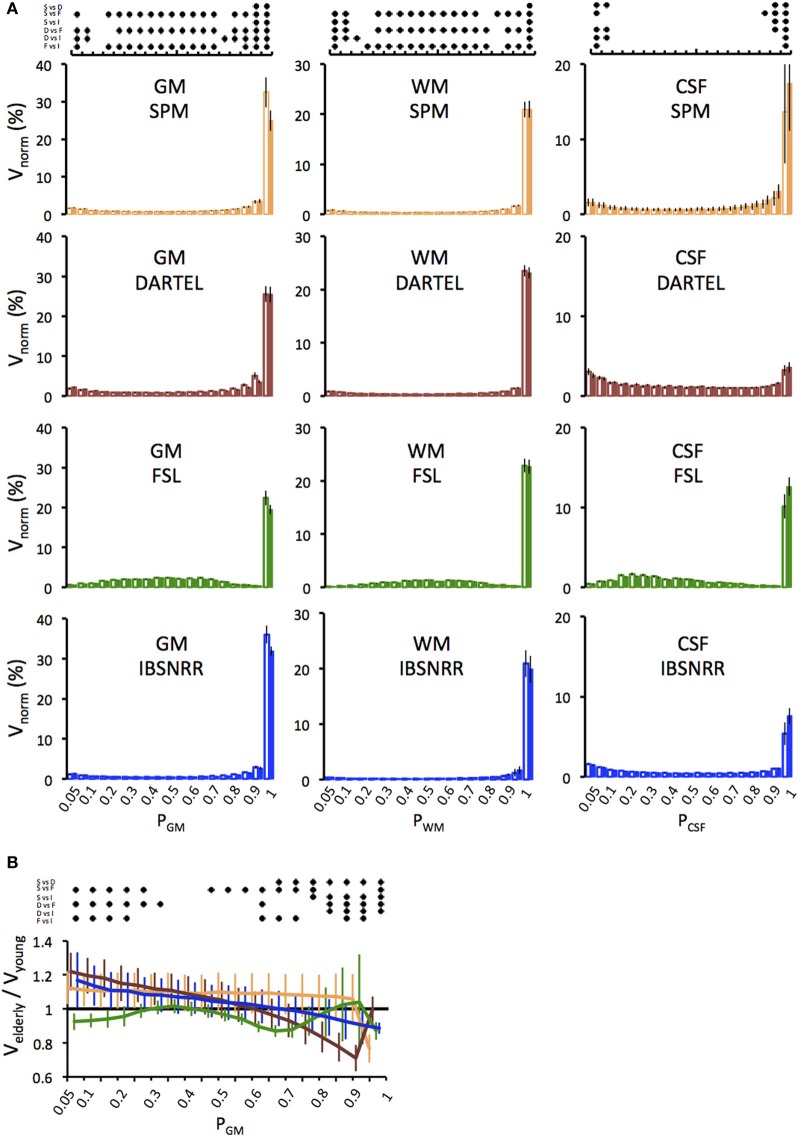
**(A)** Distributions of GM, WM and CSF probabilities in the young (open bars) and the elderly group (filled bars). Volumes per bin were normalized to ICV (V_norm_) and expressed as a percentage. Distributions were derived from the segmentation solutions provided by the unified segmentation/normalization of SPM5/SPM8 (upper panel), DARTEL (second panel), FSL (third panel) and IBSNRR (lower panel). Results of the statistics are symbolized by the dot diagram on top. For each bin, Tukey *post-hoc* tests, i.e., pairwise comparisons between all methods, were performed and significant results (*p* < 0.05) were indicated by a dot (S, SPM; D, DARTEL; F, FSL; I, IBSNRR). **(B)** Age specific differences in the GM probability distribution are shown for each method (SPM: yellow, DARTEL: red, FSL: green, ISBNRR: blue). For each bin, the GM volume of the elderly participants was expressed as a fraction of the mean volume of the young group. Significant between-method differences as tested by Tukey *post-hoc* tests (*p* < 0.05) are expressed by the dot diagram above the graph.

One might be concerned that age related effects on brain volume might be driven by partial volume effects, i.e., by voxels with a relatively low probability of belonging to a given tissue class. Therefore we determined the distributions of GM, WM and CSF probabilities as derived by either method and displayed them separately for each age group (Figure 2A). This analysis revealed several insights: First, for all tissue classes and all segmentation methods, by far the largest amount of voxels has a probability of *p* ≥ 0.9 (except for CSF derived by DARTEL). Second, distributions differ between segmentation methods. For example, with the FSL segmentation, more voxels fall into the probability range between 0.2 and 0.8 as compared to the other methods that use prior probability maps. Third, statistics revealed significant Bin × Meth interactions. In particular, all methods differ significantly from each other regarding the number of voxels assigned *p* > 0.95, and this result was found for all tissue classes.

Next we focused on GM estimates and determined how the age effect is influenced by different segmentation methods (Figure 2B). Figure 2B shows GM volume of the elderly group as a fraction of the GM volume of the young. Thus, values below 1 indicate that elderly have less voxels than the average of the young group within a given probability bin, while values larger than 1 indicate that elderly have more voxels. Again, FSL differed from all other methods, as it revealed consistently lower GM volumes in the elderly than in the young for nearly all probability ranges. By contrast, SPM, DARTEL, and IBSNRR segmentation suggested a shift of GM distribution with age, such that the elderly had a relative larger number of voxels with low GM probabilities and a relatively lower number of voxels with high GM probabilities than the young. Statistics revealed a significant Bin × Meth interaction further supporting our observation (see Figure 2 for statistics summary).

In summary, it is unlikely that age specific differences in GM volume are driven by voxels with relatively low GM probabilities. Instead, SPM, FSL, and IBSNRR segmentation find pronounced volume differences between elderly and young for voxels with high GM probability (*p* ≥ 0.9). Only DARTEL differs in this respect since the GM reduction in the elderly was mainly observed for voxel probabilities ranging between 0.6 and 0.9

### GM volumetry: interactions between age and segmentation method

**Figure 3:**
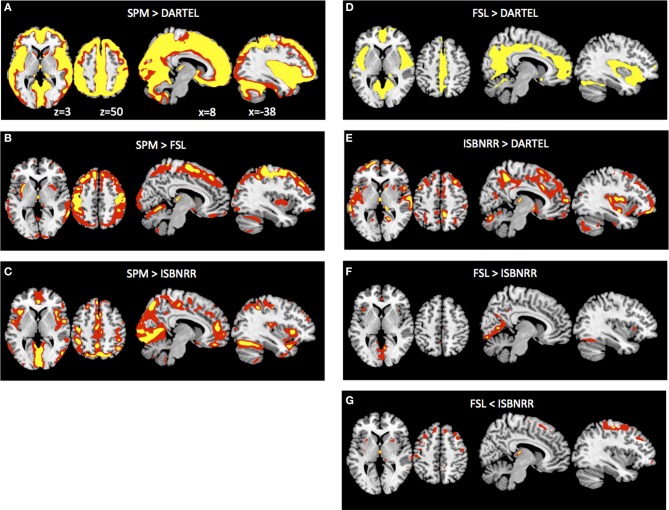
**Method × Age interaction of GM probabilities at the voxel level after common normalization to MNI space (see Methods section for details)**. Voxels exhibiting significantly larger GM differences between young versus elderly group when analyzed with SPM than with DARTEL **(A)**, than with FSL **(B)** and than with ISBNRR **(C)**. Also, FSL **(D)** and ISBNRR **(E)** revealed for some voxels larger GM differences between young versus elderly subjects than DARTEL. There were only minor differences in age-specific GM decline between FSL and ISBNRR **(F,G)**. Statistical parametric maps were either thresholded at *p* < 0.001 uncorrected (red) or *p* < 0.05 FWE corrected (yellow).

After previous analyses have established that the different segmentation methods have a significant influence on quantifying age specific changes in overall GM volumes, we asked where these method-specific differences are located in the brain. To this end we transformed all GM probability maps from native space to ICBM152 space and, importantly, applied for each individual the same transformation parameters to all GM probability maps. Subsequently we determined the Age × Meth interaction. Note that this will not reveal general differences in GM probability across methods or areas exhibiting GM decline with age. Instead this analysis was performed to identify where in the brain the age related GM decline is over- or under-estimated when compared across segmentation methods. Comparing SPM to DARTEL segmentation, SPM revealed significantly larger GM differences between young and elderly throughout the surface of the brain (Figure 3A). When SPM was compared to FSL, differences in the aging effect were most pronounced for dorsal cortical region, particularly around the central sulcus (Figure 3B). By contrast when comparing SPM to IBSNRR (Figure 3C), differences tended to be located more inferiorly, for example around the sylvian fissure. DARTEL segmentation revealed also a significantly smaller age effect on GM when compared to FSL (Figure 3D) and IBSNRR (Figure 3E): differences with both methods were particularly located around the interhemispheric and the sylvian fissure even though this effect was much more pronounced when compared to FSL than to IBSNRR. Finally, also FSL and IBSNRR segmentation exhibited minor differences when quantifying age related GM decline, such that FSL tended to reveal larger age effects for inferior located cortical surface areas (particularly around the sylvian fissure, Figure 3F) and lower age related effects for the dorsal cortical areas (Figure 3G). In summary, method specific differences seem to arise mainly from differential segmentation results for the cortical surface and particularly close to large sulci and fissures. By contrast, differences were only minor for subcortical gray matter structures.

One has to keep in mind though, that GM segmentations were transformed from native to ICBN152 space. Even though this transformation was identical across segmentation methods, it differed across subjects and mis-registrations might have exaggerated or diminished age related effects at the voxel level. Therefore, we performed a complementary ROI-based analysis for the anatomical structures predefined in the IBSNRR atlas. This analysis used GM estimates for large anatomical structures and is performed in native space. GM differences were estimated by independent *t*-tests calculated for each ROI and segmentation method. *T*-values and effect sizes are reported in Table 2. It can be seen that SPM revealed significantly lower GM volume for elderly than for young for nearly all cortical ROIs containing large surface area and that the size of these effects was usually large (> 0.08). FSL and IBSNRR revealed age related effects for frontal lobe, cingulum, insula, and parietal lobe, but less so for temporal and occipital ROIs. Moreover, the size of the age related effects were often smaller than when estimated based on SPM segmentations. For the DARTEL segmentations none of the cortical ROIs reach statistical significance. However, this might be due to our very strict correction for multiple comparisons. When inspecting effect sizes, also the DARTEL segmentation revealed large age related effects regarding GM loss in frontal and parietal lobe (Cohen's *d* > 0.95), as well as for nucleus accumbens and caudate nucleus.

**Table 2:** **Region of interest analysis based in the IBSNRR labels**.

**Region of interest**	***t*-values**				**Cohen's *d* effect size**			
	**SPM**	**DARTEL**	**FSL**	**IBSNRR**	**SPM**	**DARTEL**	**FSL**	**IBSNRR**
Frontal lobe	***−5.55857***	−2.94046	***−4.21646***	***−3.84109***	***1.822***	***0.950***	***1.383***	***1.250***
Insula	***−4.78938***	−2.08673	***−4.23940***	***−3.44408***	***1.550***	0.668	***1.372***	***1.109***
Anterior cingulum	***−4.94150***	−2.26753	***−4.93023***	***−3.52844***	***1.589***	0.723	***1.590***	***1.134***
Posterior cingulum	***−4.30735***	−2.45042	***−4.46508***	−3.24345	***1.397***	0.789	***1.450***	***1.048***
Parietal lobe	***−5.97821***	−3.23273	***−4.61079***	***−4.20736***	***1.961***	***1.044***	***1.508***	***1.373***
Superior temporal gyrus	***−3.90535***	−2.02391	***−3.59233***	−2.72604	***1.275***	0.656	***1.174***	***0.887***
Medial anterior temporal lobe	−1.58013	−0.13167	−1.33647	−0.44668	0.515	0.043	0.434	0.145
Lateral anterior temporal lobe	−1.60053	0.14597	−1.02659	−0.90367	0.523	0.047	0.334	0.293
Parahippocampal ambient gyri	***−3.80909***	−2.08526	***−3.26580***	−2.12464	***1.232***	0.669	***1.051***	0.682
Hippocampus	−0.87600	0.19980	−1.30167	−0.09042	0.279	0.064	0.420	0.029
Amygdala	0.27105	0.87557	−0.34465	0.79521	0.088	0.284	0.113	0.258
Middle inferior temporal gyri	***−3.68834***	−2.06977	−2.65204	−3.19306	***1.188***	0.661	***0.850***	***1.024***
Posterior temporal lobe	***−4.10874***	−1.70954	−3.10292	−2.29486	***1.337***	0.551	***1.010***	0.742
Lateral Occipitotemporal gyrus	***−3.36714***	−2.09976	−3.22831	−2.14540	***1.079***	0.669	***1.035***	0.684
Occipital lobe	***−3.79263***	−1.63718	−2.72239	−1.54889	***1.230***	0.527	***0.882***	0.500
Caudate nucleus	***−4.95242***	−2.78140	−2.99351	***−4.08593***	***1.611***	***0.899***	***0.959***	***1.322***
Putamen	−2.98465	−0.96431	0.18296	***−4.02995***	***0.960***	0.307	0.058	***1.276***
Nucleus accumbens	***−3.27467***	−3.23460	***−3.52531***	−3.20882	***1.044***	***1.032***	***1.149***	***1.022***
Pallidum	2.82448	5.30998	5.77498	0.58041	0.900	1.673	1.791	0.185
Thalamus	−2.72270	0.10327	1.40721	−2.33825	***0.885***	0.033	0.443	0.750
Cerebellum	−3.22701	−0.95889	−1.96225	−1.99268	***1.042***	0.307	0.636	0.640

T-values and effect sizes are reported for the comparison of GM volume between elderly and young subjects (negative t-values indicate larger volumes for the young than for the elderly). Significant results as well as effect sizes >0.8 are highlighted in bold italics.

In summary, when age related GM decline is used as a model to compare different segmentation methods, SPM is the most liberal approach revealing the largest GM differences, whereas DARTEL revealed the most conservative results (i.e., smallest effect size). This difference is particularly pronounced for cortical surface areas neighboring large fissures or large sulci.

**Figure 4:**
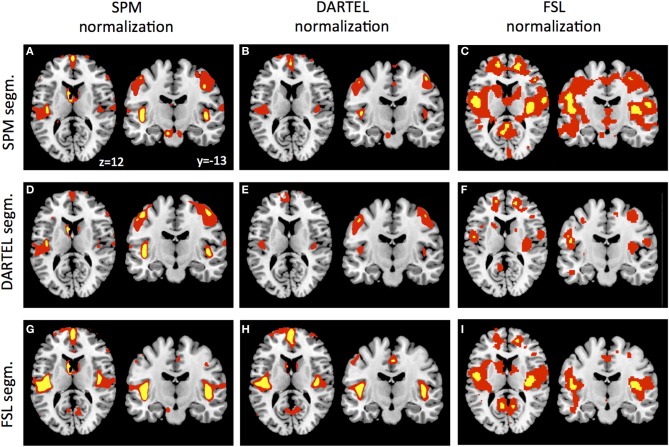
**GM segmentations derived by SPM (A–C), DARTEL (D–F), and FSL (G–I) were normalized using the warping and modulation parameters of SPM (A,D,G), DARTEL (B,E,H) and FSL (C,F,I)**. Statistical parametric maps were either thresholded at *p* < 0.001 uncorrected (red) or *p* < 0.05 FWE corrected (yellow).

As a final control, we warped the GM segmentations to ICBM152 space using the warpings derived with SPM5/SPM8, DARTEL, and FSL. Figure 4 shows the VBM results in form of statistical parametric maps when GM differences are contrasted between the young and the elderly sample. Even though this analysis reveals purely qualitative information, it is apparent that the general results pattern is similar, but also that some differences persist after normalization, particularly when more stringent statistical thresholding is used. Figures 4A,E,I show the results revealed by the SPM5/SPM8 VBM, DARTEL VBM, and FSL VBM pipeline, respectively. Representative slices show that all methods reveal age-related differences around large sulci, like the lateral fissure, but that extent and peak location differ. In accordance to the segmentation results, DARTEL VBM revealed less age-related GM differences than the other methods. SPM5/SPM8 VBM and FSL VBM revealed age-related changes of similar extent but results of FSL VBM were located more medially and superior to those of SPM5/SPM8 VBM. Furthermore, we applied each normalization method to each segmentation result. Overall, SPM5/SPM8 normalization (Figures 4A,D,G) seems to further enhance age-related differences in comparison to DARTEL (Figures 4B,E,H) while the FSL provides an intermediate solution, particularly when thresholded at pFWE < 0.05 (note though that the FSL developers advise to use non-parametric statistics for VBM analyses). In summary, when age-related decline is estimated with VBM, method specific differences persist also after normalization.

## Discussion

VBM is a largely automated approach to quantify structural changes of the brain and an interesting tool for studying large populations. In the present study we asked whether cross-sectional VBM studies are significantly influenced by segmentation and normalization algorithms provided by different software packages frequently used by the neuroimaging community. We used structural changes due to aging as a well-established model of pronounced GM reduction particularly in the frontal lobe. Our findings show that mainly segmentation but also normalization procedures have a profound influence on VBM results. Overall age-specific differences in GM estimates were largest using standard SPM VBM as implemented in SPM5/SPM8 and smallest using DARTEL VBM. Even with a rather small sample as tested here, differences between methods reached significance which is important to consider when comparing studies that used different software packages for VBM analyses.

### Ageing effects of total GM, WM, and CSF volume

Irrespective of which segmentation method was used, we found lower GM and higher CSF volume in the elderly as compared to the young (after correction for ICV). This result was highly expected based on previous literature (Good et al., 2001; Van Laere and Dierckx, 2001; Resnick et al., 2003; Tisserand et al., 2004; Grieve et al., 2005; Lemaıtre et al., 2005; Lehmbeck et al., 2006; Kalpouzos et al., 2009; Kennedy et al., 2009). However, regarding GM volume, we found large differences across segmentation methods when quantifying the effect size of the age-related decline: SPM tended to over-estimate age specific differences, while DARTEL tended to under-estimate these structural changes when compared to the other methods. This pronounced difference between SPM5/SPM8 and DARTEL segmentation has been reported before based on a much larger sample including data across the lifespan (see supplementary material in Peelle et al., 2012). One has to note however, that we tested two groups at the extreme end of the age range and this selection contributed to the large difference between SPM and DARTEL segmentation since the analysis of Peelle et al. (2012) indicated that the methodological differences regarding GM volume estimation are smaller when less extreme age groups are compared. Our analysis suggests that methods using a-priory probability maps during the segmentation algorithm (i.e., SPM, DARTEL, and IBSNRR) revealed a slightly shifted distribution of GM probabilities in the elderly: in the elderly we found more voxels with low GM probability and less with high GM probability than in the young. However, the largest part of the volume loss was driven by a profound reduction of high GM probability voxels.

All approaches revealed an increased CSF volume in the elderly vs. young subjects. SPM5/SPM8 segmentation however revealed distinctly more variable and notably higher CSF estimates than the other methods. This large CSF overestimation generated by SPM5/SPM8 is possibly derived from misclassification of non-CSF voxels in the subarachnoid space (within the dura, Mueller et al., 1998). Although CSF volume is generally not at the core interest of VBM studies, this overestimation might indirectly influence the results when incorporated for the calculation of a covariate such as ICV (Buckner et al., 2004). Typically this covariate is required when assessing tissue volumes relative to global brain volume and the advised method is to sum up thresholded GM, WM and CSF probability maps. One common workaround is to approximate total intracranial volume by summing GM and WM volume whilst omitting CSF or by using other covariates depending on which aspect of age related GM changes is investigated (Peelle et al., 2012).

### The influence of different segmentation methods on VBM

We showed that method specific differences resulted from the segmentation and were particularly pronounced for the cortical surface close to large sulci and fissures. The largest differences were found between SPM5/SPM8 and DARTEL whereas FSL and IBSNRR segmentation resulted in intermediate sensitivity regarding age-related GM changes. Method specific deviations were not only observed for voxel wise comparisons but also when anatomical regions of interest were used.

SPM5/SPM8, DARTEL, and IBSNRR made use of prior probability maps and our results suggest that segmentation depends critically on how well the anatomical image of the individual subject aligns with this prior information. Warping between the individual anatomy and a template requires non-linear, local deformations particularly around ventricles, sulci and fissures that are typically enlarged in elderly (Blatter et al., 1995; Mu et al., 1999; Raz et al., 2004; Resnick et al., 2007). This presents a challenge for normalization procedures. Comparing the unified segmentation/normalization algorithm provided by SPM5/SPM8 to DARTEL suggests that DARTEL provides a better normalization for deviant anatomy than the unified segmentation/normalization algorithm provided by SPM5/SPM8. SPM5/SPM8 results may be additionally biased because prior tissue probability maps were based on a sample consisting of relatively young, healthy subjects whereas DARTEL probability maps provide a better representation of brain anatomy across the lifespan.

One important difference between the SPM5/SPM8 and the DARTEL procedure is that the latter creates a custom template based on the population under study, thus minimizing spatial transformation across the sample. In the past it has been argued that the unified segmentation/normalization algorithm of SPM5/SPM8 eliminates the necessity for a custom template, even for anatomically deviant populations (Thomas et al., 2009). However, it might be advisable to use a customized template when extreme age-groups are compared, as in our study.

IBSNRR revealed age-related differences that were smaller than the SPM5/SM8 solution but larger than the DARTEL solution. These inconsistencies result most likely from different warping algorithms since IBSNRR uses an integrated segmentation and registration on the basis of a different deformation model, allowing larger local non-linear deformations (more degrees of freedom) than SPM5/SPM8. In agreement with our results, Klein et al. (2009) have argued that normalization solutions tend to be better when methods allow more degrees of freedom of the deformation. Alternatively, advanced segmentation methods can be used to avoid an age–related bias (Ziegler et al., 2012).

Moreover, IBSNRR uses an atlas that was created by a non-linear registration procedure so that the resulting probability maps contained much anatomical detail regarding cortical sulci (see Figure 1 for a comparison with the SPM5/SPM8 GM probability map).

FSL VBM was the only method that revealed a segmentation solution that did not rely on prior tissue probability maps and age-related GM differences were very similar to those revealed by IBSNRR. However, when FSL normalization was qualitatively compared to SPM5/SPM8 and DARTEL normalization, localization of age-related GM differences was slightly different. This is one possible explanation why some VBM results obtained with SPM5/SPM8 (Draganski et al., 2004) could not be reproduced when FSL was used (Scholz et al., 2009), even though these studies tested longitudinal rather than cross sectional GM changes.

In summary, differences in segmentation algorithms (that often rely on an initial spatial normalization step) seem to be a major source for between-method differences when VBM is used to compare the brain structure of young vs. elderly subjects (Courchesne et al., 2000; Bookstein, 2001; Davatzikos, 2004). We suggest that this effect is particularly pronounced when subjects have deviant anatomy (as it is typically the case for elderly individuals) and normalization employs only limited degrees of freedom for local deformations. Therefore, differences in the normalization algorithm might significantly influence the segmentation step when prior tissue probability maps are used, but also the localization of significant GM differences when individuals are compared within a common template space.

## Limitations of the study

In this study we investigated whether different VBM procedures have a significant influence on GM estimates of young vs. elderly subjects. Even though we found substantial differences between methods we cannot infer which VBM method is the most correct one since it is difficult to determine the ground truth for voxel-based methods. Typically this important step for method validation is done by the developers and published when a new method is introduced (see for example Ashburner and Friston, 2011). However, it is rarely the case that previous datasets are reanalyzed what makes comparison across different studies difficult. Here we report that age-specific GM differences are substantial across methods and should be considered when different findings are compared. This is particularly important when early VBM studies are considered which were mostly performed using SPM5/SPM8. Consequently, a-priory hypotheses should be formulated with care considering that SPM5/SPM8 VBM tends to overestimate age-related GM decline in relation to DARTEL, particularly when groups deviate from the anatomical SPM5/SPM8 templates and probability maps.

Another caveat is the fact that our sample was relatively small and drawn from the extremes of the age spectrum. Method specific differences are probably less pronounced when a smaller age range is considered (Peelle et al., 2012). Finally, we applied parametric statistics whilst non-parametric approaches might be generally more appropriate for VBM studies (Douaud et al., 2006). One has to keep in mind, though, that we were not interested in reporting age-specific GM differences *per se*, but rather whether these results depended on the algorithms provided by software packages frequently used by the neuroimaging community.

## Conclusion

Morphometric measurements are increasingly applied for the detection of GM changes in healthy ageing as well as in neurodegenerative disease. Due to its automated and near hypothesis-free character, VBM has gained popularity as a substitute for manual demarcations of GM within volumes of interest. Here, we used the aging brain as a well-known model for structural atrophy and asked whether comparing GM between young and elderly by VBM depends on methodological differences between commonly used software packages, i.e., the unified segmentation/normalization procedure provided by SPM5/SPM8, DARTEL, and FSL. We showed that VBM revealed the largest age-related GM differences when using the SPM5/SPM8 and the smallest when using DARTEL for segmentation. These method-specific differences reached significance when tested at various levels of description (total brain volume, regions of interest, voxel based). We argue that the segmentation procedure can have a major influence on cross-sectional VBM results, particularly when anatomical deviations are more outspoken in one group than the other. Methods that provide only limited degrees of freedom for local deformations (such as SPM5/SPM8) might overestimate between-group differences in VBM results. This is important when results are compared across studies using different VBM methods and particularly when a-priori hypotheses are derived from early VBM studies that were often performed using SPM5/SPM8 segmentation.

### Conflict of interest statement

The authors declare that the research was conducted in the absence of any commercial or financial relationships that could be construed as a potential conflict of interest.
