# Supplementary material for: Assessing age-related gray matter decline with voxel-based morphometry depends significantly on segmentation and normalization procedures
Source: Front Aging Neurosci. 2014 Jun 23;6:124. doi: 10.3389/fnagi.2014.00124 (PMC4066859; doi:10.3389/fnagi.2014.00124)
Supplement: Supplementary file 1 [file DataSheet1.PDF]

Supplementary Table 1: Method specific differences in etimates of the intracranial volume (ICV)

ICV (ml) was calculated by adding GM, WM and CSF probability maps, thresholding the result at p=0.2 and integrating the volume across all remaining voxels for each of the four different methods. However, repeated measures ANOVA with the within factor METH (SPM, DARTEL, FSL, IBSNRR) and the between factor GROUP (young, old) revealed no significant main effect of GROUP ($F(1,37)=0.009$, $p=.927$) but there were significant differences between the four methods (see below, $F(3,111)=177.97$, $p<0.0001$). To avoid a method specific bias, the diluted brain mask of the ICBM152 template, available from the FSL software package was transformed to native subject space based on the inverted, non-linear warping parameters derived from the FSL normalization procedure. The ICV was then estimated by integrating the volume across all non-zero voxels of the mask in native space. Additional correlation analyses revealed that ICV correlated significantly with the ICV determined by each of the other methods and revealed the highest r-values when compared to ICV_{DARTEL}, ICV_{FSL} and ICV_{IBSNRR} (all >0.95). Correlation with ICV_{SPM} was generally lower (around r=0.7) probably because ICV is inflated due to a documented overestimation of CSF with SPM segmentation.

Method	young	elderly	Pearson r
brain mask	1707±152.8	1707±169.9	
SPM	1770±168.7	1794±199.4	0.7167
DARTEL	1563±127.6	1585±157.4	0.9688
FSL	1454±125.5	1435±133.3	0.9581
IBSNRR	1492±137.9	1482±147.8	0.9682
